# Supplementary material for: Genome-Wide Analysis Reveals Chitinases as Putative Defense-Related Proteins Against Fungi in the Genomes of Coffea arabica and Its Progenitors
Source: Plants (Basel). 2025 Oct 10;14(20):3130. doi: 10.3390/plants14203130 (PMC12567117; doi:10.3390/plants14203130)
Supplement: Supplementary file 1 [file plants-14-03130-s001.zip › Table S8.pdf]

Table S8. Functionally characterized plant chitinase genes/proteins involved in resistance to fungal diseases in plants, based on a literature review of Chen et al, 2024 [1].

Source species	Gene/Protein ID	GH	Class	Annotation	Recipient species	Target fungus	References
Bitter melon	DQ407723.1<br>ABD66068.1	19	I	Mcchit1	Rice	*Magnaporthe grisea*<br>*Rhizoctonia solani*	[2]
					Cotton	*Verticillium dahliae*	[3]
Barley	AJ276226.1<br>CAB99486.1	19	II	Chi2	Potato	*Alternaria solani*	[4]
	AAA56786.1	19	II	CHI	Blackgram	*Corynespora cassiicola*	[5]
					Tobacco	*Rhizoctonia solani*	[6]
	M62904.1<br>AAA32941.1	19	II	Chi26	Wheat	*Fusarium graminearum*	[7]
						Puccinia recondite<br>*Puccinia striiformis* f. sp. tritici<br>*Blumeria graminis*	[8]
	KC899774.1<br>AGS38341.1	19	II	CEMB-chiII	Sugarcane	*Colletotrichum falcatum*	[9]
Wheat	AAD28730	19	VII	Chi194	Tomato	*Fusarium oxysporum* f. sp. Lycopersici	[10]
Bean	S43926.1<br>AAB23263.1			Chi<br>CH5B	Cotton	*Verticillium dahlia*	[11]
					Canola	*Rhizoctonia solani*	[12]

					Tobacco	*Rhizoctonia solani*	[13]
					Strawberry	*Botrytis cinerea*	[14]
Chinese wild strawberry	MN709779 QLY89005.1	18	V	FnCHIT2	*Arabidopsis*	*Colletotrichum higginsianum*	[15]
Cacao	U30324 AAA80656.1	19	I	TcChi1	Cacao	*Colletotrichum gloeosporioides*	[16]
Cucumber	NM_001308904.2 NP_001295833.1	18	III	CHI2	Cucumber	*Botrytis cinerea*	[17]
Eucommia ulmoides	KJ413009.1 AHX74093.1	19	I	EuCHIT2	Tobacco	*Erysiphe cichoracearum*	[18]
Apple	LOC103401024 NP_001280823.1	19	II	MdCHI1	Apple	*Colletotrichum gloeosporioides Alternaria alternata*	[19]
Brassica juncea	EF586206 ABQ57389.1	19	IV	Bj chitinase IV	*Brassica juncea*	*Alternaria brassica*	[20]
Mulberry	EXB55192.1	19	IV	MnChi18	*Arabidopsis*	*Botrytis cinerea*	[21]
Maize	MG017374.1 AYK28286.1	19	I	Chit2	Corn	*Fusarium graminearum*	[22]
Capsicum annuum	KJ649334.1 AJF11981.1	19	IV	CaChitIV	Arabidopsis	*Hyaloperonospora arabidopsidis*	[23]
Rice	LOC_Os03g30470 XP_015629397.1			RCH10	Rose	*Diplocarpon rosae*	[24]

					Lilium	*Botrytis cinerea*	[25]
	LOC_Os05g33130<br>XP_015640432.1	19	I	Chitinase2<br>Cht-2<br>RCC2<br>RCG3<br>RC7<br>ChtBD1<br>RC24	Banana	*Mycosphaerella fijiensis*	[26]
					Chrysanthemum	*Botrytis cinerea*	[27]
					Cucumber	*Botrytis cinerea*	[28]
					Cucumber	*Botrytis cinerea*	[29]
					Grapevine	*Uncinula necator*	[30]
					Italian ryegrass	*Puccinia coronata*	[31]
					Indica Rice	*Rhizoctonia solani*	[32]
					Peanut	*Cercospora arachidicola*	[33]
					Rice	*Magnaporthe grisea*	[34]
					Strawberry	*Sphaerotheca humuli*	[35]
					Tomato	*Alternaria solani*<br>*Fusarium oxysporum* f. sp. Lycopersici	[36]

					Wheat	*Puccinia striiformis* f. sp. Tritici	[37]
	X54367.1 CAA38249.1	19	I	Chil1 RCC11 Rchit	Finger millet	*Pyricularia grisea*	[38]
					Grapevine	*Uncinula necator*	[39]
					Litchi	*Phomopsis* sp.	[40]
					Peanut	Aspergillus flavus	[41]
					Rice	*Rhizoctonia solani*	[42]
	LOC_Os11g47510 ABA95474.1	18			Rice	*Rhizoctonia solani*	[43]
Roundleaved Sundew	KU516826.1 AMM76171.1	19	I	DrChit	Tobacco	*Trichoderma viride*	[44]
Strawberry	OQ211094.1 WGF83129.1	19	II	FvChi-14	*Arabidopsis*	*Colletotrichum higginsianum*	[45]
Sweet potato	MN971588.1 QOD94995.1	19	II	IbChiA	Sweet potato	*Ceratocystis fimbriata*	[46]
Sugarbeet	A23392.1 CAA01677.1	19	IV	Chitinase IV	Silver birch	*Melampsoridium botulinum*	[47]
						Pyrenopeziza betulicola	[48]

Tobacco	X16938.1<br>CAA34812.1	19	I	Tob<br>CHI	Tobacco	*Rhizoctonia solani*	[49]
					Peanut	*Cercospora arachidicola*	[50]
Wild rice	EU850802.1<br>ACJ24349.1	19	IV	OgChitIVa	*Arabidopsis*	*Botrytis cinerea*	[51]
Wild tomato	LOC107008831<br>XP_015063508.1			pcht28	Strawberry	*Verticillium dahliae*	[52]
					Tomato	*Verticillium dahliae* race 2	[53]

References

1. Chen, J.-Y.; Sang, H.; Chilvers, M.I.; Wu, C.-H.; Chang, H.-X. Characterization of soybean chitinase genes induced by rhizobacteria involved in the defense against *Fusarium oxysporum*. *Frontiers in Plant Science* **2024**, *15*, 1341181. https://doi.org/10.3389/fpls.2024.1341181
2. Li, P.; Pei, Y.; Sang, X.; Ling, Y.; Yang, Z.; He, G. Transgenic indica rice expressing a bitter melon (*Momordica charantia*) class I chitinase gene (McCHIT1) confers enhanced resistance to *Magnaporthe grisea* and *Rhizoctonia solani*. *European Journal of Plant Pathology* **2009**, *125*, 533–543. https://doi.org/10.1007/s10658-009-9501-8
3. Xiao, Y.-H.; Li, X.-B.; Yang, X.-Y.; Luo, M.; Hou, L.; Guo, S.-H.; Luo, X.-Y.; Pei, Y. Cloning and characterization of a balsam pear class I chitinase gene (McCHIT1) and its ectopic expression enhances fungal resistance in transgenic plants. *Bioscience, Biotechnology, and Biochemistry* **2007**, *71*, 1211–1219. https://doi.org/10.1271/bbb.60658
4. Khan, A.; Nasir, I.A.; Tabassum, B.; Aaliya, K.; Tariq, M.; Rao, A.Q. Expression studies of chitinase gene in transgenic potato against *Alternaria solani*. *Plant Cell, Tissue and Organ Culture* **2017**, *128*, 563–576. https://doi.org/10.1007/s11240-016-1134-y
5. Chopra, R.; Saini, R. Transformation of blackgram (*Vigna mungo* (L.) Hepper) by barley chitinase and ribosome-inactivating protein genes towards improving resistance to *Corynespora* leaf spot fungal disease. *Applied Biochemistry and Biotechnology* **2014**, *174*, 2791–2800. https://doi.org/10.1007/s12010-014-1226-2
6. Jach, G.; Görnhardt, B.; Mundy, J.; Logemann, J.; Pinsdorf, E.; Leah, R.; Schell, J.; Maas, C. Enhanced quantitative resistance against fungal disease by combinatorial expression of different barley antifungal proteins in transgenic tobacco. *The Plant Journal* **1995**, *8*, 97–109. https://doi.org/10.1046/j.1365-313X.1995.08010097.x
7. Shin, S.; Mackintosh, C.A.; Lewis, J.; Heinen, S.J.; Radmer, L.; Dill-Macky, R.; Baldridge, G.D.; Zeyen, R.J.; Muehlbauer, G.J. Transgenic wheat expressing a barley class II chitinase gene has enhanced resistance against *Fusarium graminearum*. *Journal of Experimental Botany* **2008**, *59*, 2371–2378. https://doi.org/10.1093/jxb/ern103
8. Eissa, H.F.; Hassanien, S.E.; Ramadan, A.M.; El-Shamy, M.M.; Saleh, O.M.; Shokry, A.M.; Abdelsattar, M.; Morsy, Y.B.; El-Maghraby, M.A.; Alameldin, H.F.; et al. Developing transgenic wheat to encounter rusts and powdery mildew by overexpressing barley Chi26 gene for fungal resistance. *Plant Methods* **2017**, *13*, 41. https://doi.org/10.1186/s13007-017-0191-5
9. Tariq, M.; Khan, A.; Tabassum, B.; Toufiq, N.; Bhatti, M.U.; Riaz, S.; Nasir, I.A.; Husnain, T. Antifungal activity of chitinase II against *Colletotrichum falcatum* Went. causing red rot disease in transgenic sugarcane. *Turkish Journal of Biology* **2018**, *42*, 45–53. https://doi.org/10.3906/biy-1709-17
10. Girhepuje, P.V.; Shinde, G.B. Transgenic tomato plants expressing a wheat endochitinase gene demonstrate enhanced resistance to *Fusarium oxysporum* f. sp. *lycopersici*. *Plant Cell, Tissue and Organ Culture* **2011**, *105*, 243–251. https://doi.org/10.1007/s11240-010-9859-5
11. Tohidfar, M.; Mohammadi, M.; Ghareyazie, B. *Agrobacterium*-mediated transformation of cotton (*Gossypium hirsutum*) using a heterologous bean chitinase gene. *Plant Cell, Tissue and Organ Culture* **2005**, *83*, 83–96. https://doi.org/10.1007/s11240-004-6155-2
12. Benhamou, N.; Broglie, K.; Chet, I.; Broglie, R. Cytology of infection of 35S-bean chitinase transgenic canola plants by *Rhizoctonia solani*: Cytochemical aspects of chitin breakdown *in vivo*. *The Plant Journal* **1993**, *4*, 295–305. https://doi.org/10.1046/j.1365-313X.1993.04020295.x
13. Broglie, K.; Chet, I.; Holliday, M.; Cressman, R.; Biddle, P.; Knowlton, S.; Mauvais, C.J.; Broglie, R. Transgenic plants with enhanced resistance to the fungal pathogen *Rhizoctonia solani*. *Science* **1991**, *254*, 1194–1197. https://doi.org/10.1126/science.254.5035.1194

14. Vellicce, G.R.; Ricci, J.C.D.; Hernández, L.; Castagnaro, A.P. Enhanced resistance to *Botrytis cinerea* mediated by the transgenic expression of the chitinase gene *Ch5B* in strawberry. *Transgenic Research* **2006**, *15*, 57–68. https://doi.org/10.1007/s11248-005-2543-6
15. Wen, Z.; Bai, J.; Wang, L.; Yao, L.; Ahmad, B.; Hanif, M.; Chen, Q. Overexpression of a chitinase 2 gene from Chinese wild strawberry improves resistance to anthracnose disease in transgenic *Arabidopsis thaliana*. *Plant Biotechnology Reports* **2020**, *14*, 725–736. https://doi.org/10.1007/s11816-020-00648-z
16. Maximova, S.N.; Marelli, J.P.; Young, A.; Pishak, S.; Verica, J.A.; Guiltinan, M.J. Over-expression of a cacao class I chitinase gene in *Theobroma cacao* L. enhances resistance against the pathogen, *Colletotrichum gloeosporioides*. *Planta* **2006**, *224*, 740–749. https://doi.org/10.1007/s00425-005-0188-6
17. Kishimoto, K.; Nishizawa, Y.; Tabei, Y.; Nakajima, M.; Hibi, T.; Akutsu, K. Transgenic cucumber expressing an endogenous class III chitinase gene has reduced symptoms from *Botrytis cinerea*. *Journal of General Plant Pathology* **2004**, *70*, 314–320. https://doi.org/10.1007/s10327-004-0152-5
18. Dong, X.; Zhao, Y.; Ran, X.; Guo, L.; Zhao, D.G. Overexpression of a new chitinase gene *EuCHIT2* enhances resistance to *Erysiphe cichoracearum* DC in tobacco plants. *International Journal of Molecular Sciences* **2017**, *18*, 2331. https://doi.org/10.3390/ijms18112361
19. Wang, F.; Yang, S.; Wang, Y.; Zhang, B.; Zhang, F.; Xue, H.; Jiang, Q.; Ma, Y. Overexpression of chitinase gene enhances resistance to *Colletotrichum gloeosporioides* and *Alternaria alternata* in apple (*Malus × domestica*). *Scientia Horticulturae* **2021**, *277*, 109779. https://doi.org/10.1016/j.scienta.2020.109779
20. Mir, Z.A.; Ali, S.; Shivaraj, S.M.; Bhat, J.A.; Singh, A.; Yadav, P.; Rawat, S.; Paplao, P.K.; Grover, A. Genome-wide identification and characterization of chitinase gene family in *Brassica juncea* and *Camelina sativa* in response to *Alternaria brassicae*. *Genomics* **2020**, *112*, 749–763. https://doi.org/10.1016/j.ygeno.2019.05.011
21. Xin, Y.; Wang, D.; Han, S.; Li, S.; Gong, N.; Fan, Y.; Ji, X. Characterization of the chitinase gene family in mulberry (*Morus notabilis*) and *MnChi18* involved in resistance to *Botrytis cinerea*. *Genes* **2022**, *13*, 98. https://doi.org/10.3390/genes13010098
22. Dowd, P.F.; Naumann, T.A.; Price, N.P.J.; Johnson, E.T. Identification of a maize (*Zea mays*) chitinase allele sequence suitable for a role in ear rot fungal resistance. *Agri Gene* **2018**, *7*, 15–22. https://doi.org/10.1016/j.aggene.2017.10.001
23. Kim, D.S.; Kim, N.H.; Hwang, B.K. The *Capsicum annuum* class IV chitinase ChitIV interacts with receptor-like cytoplasmic protein kinase PIK1 to accelerate PIK1-triggered cell death and defence responses. *Journal of Experimental Botany* **2015**, *66*, 1987–1999. https://doi.org/10.1093/jxb/erv001
24. Marchant, R.; Davey, M.R.; Lucas, J.A.; Lamb, C.J.; Dixon, R.A.; Power, J.B. Expression of a chitinase transgene in rose (*Rosa hybrida* L.) reduces development of blackspot disease (*Diplocarpon rosae* Wolf). *Molecular Breeding* **1998**, *4*, 187–194. https://doi.org/10.1023/A:1009642707505
25. Núñez de Cáceres González, F.F.; Davey, M.R.; Cancho Sanchez, E.; Wilson, Z.A. Conferred resistance to *Botrytis cinerea* in *Lilium* by overexpression of the *RCH10* chitinase gene. *Plant Cell Reports* **2015**, *34*, 1201–1209. https://doi.org/10.1007/s00299-015-1778-9
26. Kovács, G.; Sági, L.; Jacon, G.; Arinaitwe, G.; Busogoro, J.P.; Thiry, E.; Strosse, H.; Swennen, R.; Remy, S. Expression of a rice chitinase gene in transgenic banana ("Gros Michel", AAA genome group) confers resistance to black leaf streak disease. *Transgenic Research* **2013**, *22*, 117–130. https://doi.org/10.1007/s11248-012-9631-1
27. Takatsu, Y.; Nishizawa, Y.; Hibi, T.; Akutsu, M. Transgenic Chrysanthemum (*Dendranthema grandiflorum* (Ramat.) Kitamura) Expressing a Rice Chitinase Gene Shows Enhanced Resistance to Gray Mold (*Botrytis cinerea*). *Sci. Hortic.* **1999**, *82*, 113–123. https://doi.org/10.1016/S0304-4238(99)00034-5

28. Tabei, Y.; Kitade, S.; Nishizawa, Y.; Kikuchi, N.; Kayano, T.; Hibi, T.; Akutsu, K. Transgenic cucumber plants harboring a rice chitinase gene exhibit enhanced resistance to gray mold (*Botrytis cinerea*). *Plant Cell Reports* **1998**, *17*, 159–164. https://doi.org/10.1007/s002990050371
29. Kishimoto, K.; Nishizawa, Y.; Tabei, Y.; Hibi, T.; Nakajima, M.; Akutsu, K. Detailed analysis of rice chitinase gene expression in transgenic cucumber plants showing different levels of disease resistance to gray mold (*Botrytis cinerea*) 2002, 162, 655–662. https://doi.org/10.1016/S0168-9452(01)00602-1
30. Yamamoto, T.; Iketani, H.; Ieki, H.; Nishizawa, Y.; Notsuka, K.; Hibi, T.; Hayashi, T.; Matsuta, N. Transgenic Grapevine Plants Expressing a Rice Chitinase with Enhanced Resistance to Fungal Pathogens. *Plant Cell Rep.* **2000**, *19*, 639–646. https://doi.org/10.1007/s002999900174
31. Takahashi, W.; Fujimori, M.; Miura, Y.; Komatsu, T.; Nishizawa, Y.; Hibi, T.; Takamizo, T. Increased resistance to crown rust disease in transgenic Italian ryegrass (*Lolium multiflorum* Lam.) expressing the rice chitinase gene. *Plant Cell Reports* **2005**, *23*, 811–818. https://doi.org/10.1007/s00299-004-0900-1
32. Datta, K.; Tu, J.; Oliva, N.; Ona, I.; Velazhahan, R.; Mew, T.W.; Muthukrishnan, S.; Datta, S.K. Enhanced resistance to sheath blight by constitutive expression of infection-related rice chitinase in transgenic elite indica rice cultivars. *Plant Science* **2001**, *160*, 405–414. https://doi.org/10.1016/S0168-9452(00)00413-1
33. Iqbal, M.M.; Nazir, F.; Ali, S.; Asif, M.A.; Zafar, Y.; Iqbal, J.; Ali, G.M. Overexpression of rice chitinase gene in transgenic peanut (*Arachis hypogaea* L.) improves resistance against leaf spot. *Molecular Biotechnology* **2012**, *50*, 129–136. https://doi.org/10.1007/s12033-011-9426-2
34. Nishizawa, Y.; Nishio, Z.; Nakazono, K.; Soma, M.; Nakajima, E.; Ugaki, M.; Hibi, T. Enhanced resistance to blast (*Magnaporthe grisea*) in transgenic japonica rice by constitutive expression of rice chitinase. *Theoretical and Applied Genetics* **1999**, *99*, 383–390. https://doi.org/10.1007/s001220051248
35. Asao, H.; Nishizawa, Y.; Arai, S.; Sato, T.; Hirai, M.; Yoshida, K.; Shinmyo, A.; Hibi, T. Enhanced resistance against a fungal pathogen *Sphaerotheca humuli* in transgenic strawberry expressing a rice chitinase gene. *Plant Biotechnology* **1997**, *14*, 145–149. https://doi.org/10.5511/plantbiotechnology.14.145
36. Jabeen, N.; Chaudhary, Z.; Gulfraz, M.; Rashid, H.; Mirza, B. Expression of rice chitinase gene in genetically engineered tomato confers enhanced resistance to Fusarium wilt and early blight. *The Plant Pathology Journal* **2015**, *31*, 252–258. https://doi.org/10.5423/PPJ.OA.03.2015.0026
37. Huang, X.; Wang, J.; Du, Z.; Zhang, C.; Li, L.; Xu, Z. Enhanced resistance to stripe rust disease in transgenic wheat expressing the rice chitinase gene RC24. *Transgenic Research* **2013**, *22*, 939–947. https://doi.org/10.1007/s11248-013-9704-9
38. Ignacimuthu, S.; Ceasar, S.A. Development of transgenic finger millet (*Eleusine coracana* (L.) Gaertn.) resistant to leaf blast disease. *Journal of Biosciences* **2012**, *37*, 135–147. https://doi.org/10.1007/s12038-011-9178-y
39. Nirala, N.K.; Bhattacharya, A.; Awasthi, S.; Nirala, S.; Singh, S.K.; Shukla, P.K.; Tuli, R. Expression of a Rice Chitinase Gene Enhances Antifungal Potential in Transgenic Grapevine (*Vitis vinifera* L.). *Vitis* **2010**, *49*, 181–187.
40. Das, D.K.; Rahman, A. Expression of a rice chitinase gene enhances antifungal response in transgenic litchi (cv. Bedana). *American Journal of Plant Sciences* **2018**, *9*, 2256–2275. https://doi.org/10.4236/ajps.2018.911163
41. Prasad, K.; Bhatnagar-Mathur, P.; Waliyar, F.; Sharma, K.K. Overexpression of a chitinase gene in transgenic peanut confers enhanced resistance to major soil-borne and foliar fungal pathogens. *Journal of Plant Biochemistry and Biotechnology* **2013**, *22*, 222–233. https://doi.org/10.1007/s13562-012-0155-9

42. Rajesh, T.; Maruthasalam, S.; Kalpana, K.; Poovannan, K.; Kumar, K.K.; Kokiladevi, E.; Sudhakar, D.; Samiyappan, R.; Balasubramanian, P. Stability of sheath blight resistance in transgenic ASD16 rice lines expressing a rice *Chi11* gene encoding chitinase. *Biologia Plantarum* **2016**, *60*, 749–756. https://doi.org/10.1007/s10535-016-0594-6
43. Richa, K.; Tiwari, I.M.; Devanna, B.N.; Botella, J.R.; Sharma, V.; Sharma, T.R. Novel chitinase gene LOC_Os11g47510 from indica rice 'Tetep' provides enhanced resistance against sheath blight pathogen *Rhizoctonia solani* in rice. *Frontiers in Plant Science* **2017**, *8*, 596. https://doi.org/10.3389/fpls.2017.00596
44. Durechova, D.; Jopcik, M.; Rajninec, M.; Moravcikova, J.; Libantova, J. Expression of *Drosera rotundifolia* chitinase in transgenic tobacco plants enhanced their antifungal potential. *Molecular Biotechnology* **2019**, *61*, 916–928. https://doi.org/10.1007/s12033-019-00214-1
45. He, T.; Fan, J.; Jiao, G.; Liu, Y.; Zhang, Q.; Luo, N.; Ahmad, B.; Chen, Q.; Wen, Z. Bioinformatics and expression analysis of the chitinase genes in strawberry (*Fragaria vesca*) and functional study of FvChi-14. *Plants* **2023**, *12*, 1543. https://doi.org/10.3390/plants12071543
46. Liu, M.; Gong, Y.; Sun, H.; Zhang, J.; Zhang, L.; Sun, J.; Han, Y.; Huang, J.; Wu, Q.; Zhang, C.; et al. Characterization of a novel chitinase from sweet potato and its fungicidal effect against *Ceratocystis fimbriata*. *Journal of Agricultural and Food Chemistry* **2020**, *68*, 7591–7600. https://doi.org/10.1021/acs.jafc.0c01813
47. Pasonen, H.L.; Seppänen, S.K.; Degefu, Y.; Rytkönen, A.; von Weissenberg, K.; Pappinen, A. Field performance of chitinase transgenic silver birches (*Betula pendula*): Resistance to fungal diseases. *Theoretical and Applied Genetics* **2004**, *109*, 562–570. https://doi.org/10.1007/s00122-004-1650-8
48. Pappinen, A.; Degefu, Y.; Syrjälä, L.; Keinonen, K.; von Weissenberg, K. Transgenic silver birch (*Betula pendula*) expressing sugarbeet chitinase 4 shows enhanced resistance to *Pyrenopeziza betulicola*. *Plant Cell Reports* **2002**, *20*, 1046–1051. https://doi.org/10.1007/s00299-002-0449-9
49. Vierheilig, H. Research notes: Colonization of transgenic *Nicotiana sylvestris* plants, expressing different forms of *Nicotiana tabacum* chitinase, by the root pathogen *Rhizoctonia solani* and by the mycorrhizal symbiont *Glomus mosseae*. *Molecular Plant-Microbe Interactions* **1993**, *6*, 261–264. https://doi.org/10.1094/MPMI-6-261
50. Rohini, V.K.; Sankara Rao, K. Transformation of Peanut (*Arachis hypogaea* L.) with Tobacco Chitinase Gene: Variable Response of Transformants to Leaf Spot Disease. *Plant Sci.* **2001**, *160*, 889–898. https://doi.org/10.1016/S0168-9452(00)00462-3
51. Pak, J.H.; Chung, E.S.; Shin, S.H.; Jeon, E.H.; Kim, M.J.; Lee, H.Y.; Jeung, J.U.; Hyung, N.I.; Lee, J.H.; Chung, Y.S. Enhanced fungal resistance in *Arabidopsis* expressing wild rice PR-3 (*OgChitIVa*) encoding chitinase class IV. *Plant Biotechnology Reports* **2009**, *3*, 147–155. https://doi.org/10.1007/s11816-009-0084-9
52. Chalavi, V.; Tabaeizadeh, Z.; Thibodeau, P. Enhanced Resistance to *Verticillium dahliae* in Transgenic Strawberry Plants Expressing a *Lycopersicon chilense* Chitinase Gene. *J. Am. Soc. Hortic. Sci.* **2003**, *128*, 747–753. https://doi.org/10.21273/JASHS.128.5.0747
53. Tabaeizadeh, Z.; Agharbaoui, Z.; Harrak, H.; Poysa, V. Transgenic Tomato Plants Expressing a *Lycopersicon chilense* Chitinase Gene Demonstrate Improved Resistance to *Verticillium dahliae* Race 2. *Plant Cell Rep.* **1999**, *19*, 197–202. https://doi.org/10.1007/s002990050729
